# Awake, Offline Processing during Associative Learning

**DOI:** 10.1371/journal.pone.0127522

**Published:** 2016-04-27

**Authors:** James K. Bursley, Adrian Nestor, Michael J. Tarr, J. David Creswell

**Affiliations:** 1 Department of Psychology and Center for Brain Science, Harvard University, Cambridge, Massachusetts, United States of America; 2 Department of Psychology, University of Toronto Scarborough, Toronto, Ontario, Canada; 3 Department of Psychology and Center for the Neural Basis of Cognition, Carnegie Mellon University, Pittsburgh, Pennsylvania, United States of America; National Institute of Mental Health, UNITED STATES

## Abstract

Offline processing has been shown to strengthen memory traces and enhance learning in the absence of conscious rehearsal or awareness. Here we evaluate whether a brief, two-minute offline processing period can boost associative learning and test a memory reactivation account for these offline processing effects. After encoding paired associates, subjects either completed a distractor task for two minutes or were immediately tested for memory of the pairs in a counterbalanced, within-subjects functional magnetic resonance imaging study. Results showed that brief, awake, offline processing improves memory for associate pairs. Moreover, multi-voxel pattern analysis of the neuroimaging data suggested reactivation of encoded memory representations in dorsolateral prefrontal cortex during offline processing. These results signify the first demonstration of awake, active, offline enhancement of associative memory and suggest that such enhancement is accompanied by the offline reactivation of encoded memory representations.

## Introduction

Initial studies in the learning and memory domain suggest a counterintuitive idea for improving learning, namely that following initial encoding, memory traces may be enhanced outside of conscious awareness and in the absence of any conscious rehearsal [1–3]. We propose that this post-encoding offline processing, which occurs during both sleep and wakefulness, can facilitate associative learning, and that this facilitation is accompanied by the reactivation of encoded memory representations. Here we examine whether a brief duration of two minutes is sufficient for offline processing, and whether this offline processing improves associative learning performance. Despite numerous demonstrations of offline enhancement of procedural memory [3,4], declarative memory improvement after awake, offline processing has not been investigated as widely (but see [1,5,6]), and two minutes is more than an order of magnitude shorter than durations typically used in studies of offline memory processing [2,7,8], and such timescales do not typically lead to significant memory enhancement [1]. In addition, we use multi-voxel pattern analysis (MVPA) of neuroimaging data to explore neural mechanisms of offline processing in associative learning and test for the reactivation of encoded memory representations.

Recent insights into the neural underpinnings of offline processing come from work demonstrating changes in activity [9–14] and connectivity [9,15,16] following encoding. We previously reported that reinstatement of univariate neural activations during awake, offline processing is seen in encoding-related brain regions, specifically right dorsolateral prefrontal cortex (DLPFC) and left visual cortex [9] (also see [13]). Such studies provide initial evidence that memory reactivation supports offline processing. Extending this work, the present experiment evaluates whether a brief period of offline processing improves associative memory in concert with the reactivation of memory representations during the offline processing period.

The present research employs a paired-associates learning paradigm in which subjects acquired the names and images of fictitious animals, on which they were later probed. We predicted associative memory enhancement after a cognitively demanding two-minute distractor task following encoding, compared to a condition in which no distractor task was performed and subjects were probed immediately after encoding [17]. We had two hypotheses regarding neural activity during offline processing. First, we predicted that local activity patterns in encoding-related regions, including DLPFC, would reflect the occurrence of offline processing, even when controlling for activity associated with the distractor task. DLPFC supports control and organizational processes in associative encoding and retrieval [18,19] and was implicated in our previous investigation of offline processing [9]. Second, we predicted that voxelwise patterns of activity associated with encoding in DLPFC would be reinstated during offline processing, suggesting local reactivation of encoded memory representations [20].

## Materials and Methods

### Subjects

Forty-one healthy volunteers (18 females, aged 18–35) were recruited from Carnegie Mellon University and surrounding communities in Pittsburgh, Pennsylvania. Subjects were screened based on standard MRI safety guidelines (e.g., metal implants, claustrophobia). None of the subjects had any major physical or mental health problems and did not use any psychotropic medications affecting cardiovascular or endocrine function. In addition, subjects were required to be right-handed and to speak English as their exclusive first language. Subjects gave written informed consent in accordance with the guidelines and approval of the Carnegie Mellon University Institutional Review Board prior to beginning the experiment. Each subjects was paid $25 plus a performance-based bonus of up to $12 upon completion. Of the forty-one subjects, five were excluded from analyses one the basis of excessive head movement during scans, and one was excluded for reporting unfamiliarity with more than 20% of the real animal stimuli and familiarity with more than 20% of the fictitious animal stimuli, resulting in a final sample of 35 subjects.

### Procedure

Stimuli were presented on a screen using E-Prime software (Psychology Software Tools, Pittsburgh, Pennsylvania) to subjects while they underwent functional MRI (fMRI) and made responses during tasks using a right-handed button glove. Subjects were trained on all tasks and on use of the button glove prior to entering the scanner.

**Fig 1:**
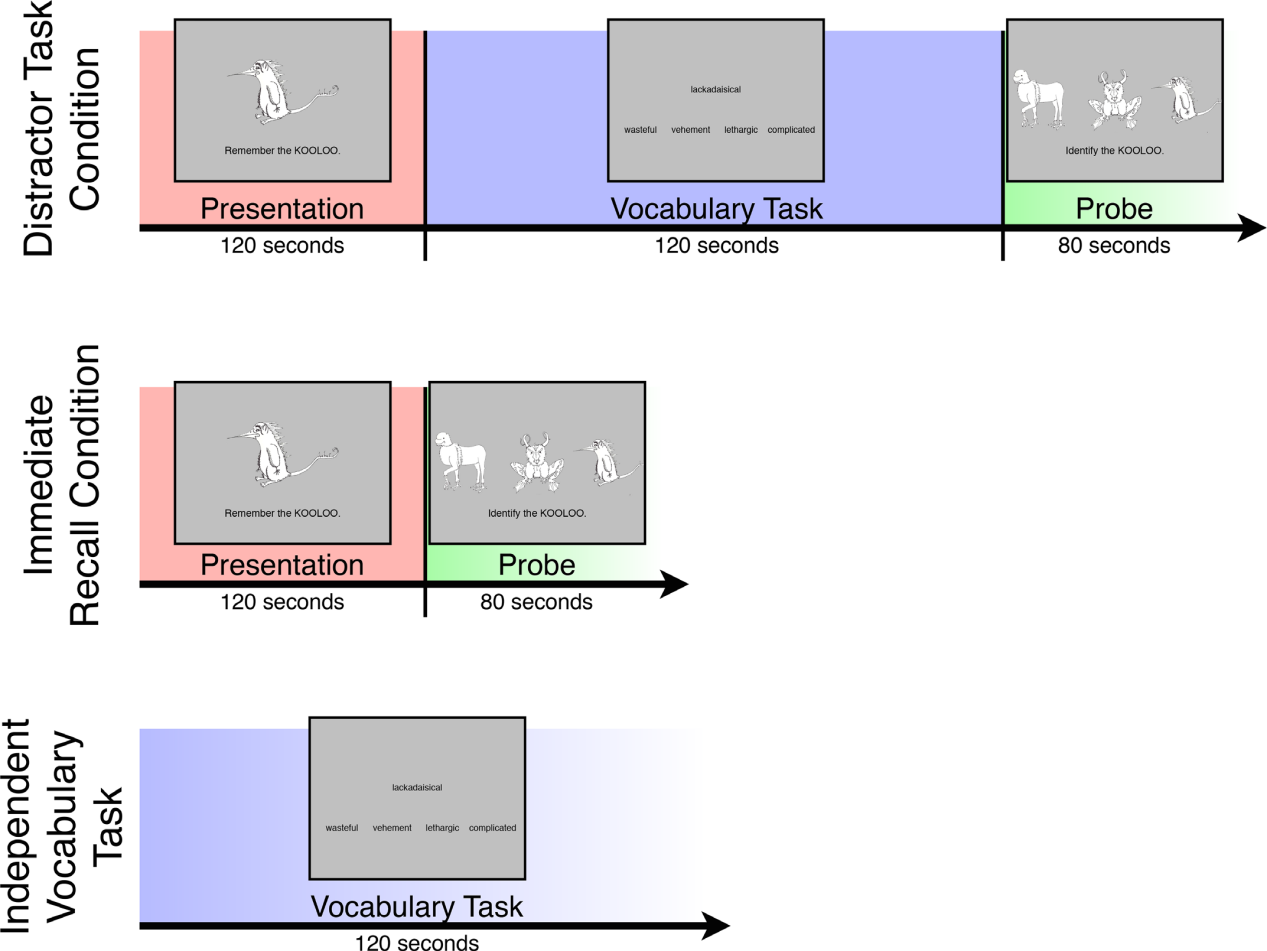
Tasks completed by subjects in the experiment. See Materials and Methods for details.

Subjects completed a paired-associates learning task (see Fig 1) adapted from [21] while undergoing fMRI. In this task, 16 images of novel, fictitious animals were presented serially on the screen for 3,750 ms each, accompanied by the instruction, “Remember the [name],” where [name] signifies a fictitious animal name (e.g., “chazbit”) randomly assigned to the presented animal. Animal image/name pairs were each presented two times in random order, with the image/name pairing remaining the same in both presentations, with the entire presentation period lasting 120 seconds. After this presentation period, subjects were assigned to either a “distractor task” condition or an “immediate-recall” condition to allow identification of neural patterns present exclusively during offline processing of animal image/name pairs. In the distractor-task condition, subjects completed a vocabulary task as a distractor for 120 seconds. In this task, a stimulus word was presented in the middle of the screen, along with four probe words below it. One of the probe words was a synonym of the stimulus word, and the remaining three were lures. Subjects were asked to indicate, via button-press, the probe word that was the synonym of the stimulus word. The stimulus and probes appeared on the screen for 8 seconds, after which they were replaced by a new stimulus/probes set. No feedback was presented during the vocabulary task.

After the vocabulary distractor task, subjects were probed for recall of the fictitious animal image/name pairs. Specifically, three of the previously presented fictitious animal images were displayed on the screen, along with the name that was paired with one of the images during the presentation period, for 5 seconds. Thus two of the animals during each probe trial were drawn randomly from the remaining presented animals, and each animal could be presented multiple times as lures during probe trials. Subjects indicated via button-press which of the animals they believed was previously paired with the name, without receiving feedback. The probe period lasted a total of 80 seconds, encompassing 16 trials, or one per presented animal. After the probe period, subjects saw a screen for 5 seconds that indicated whether or not their performance on that task had earned them a $2 bonus. Before image/name pair presentation, vocabulary tasks, and probe periods, an appropriate instruction screen (“LEARN ANIMALS,” “VOCABULARY TASK,” “IDENTIFY ANIMALS”) appeared for 5 seconds.

Subjects did not complete a distractor task during the immediate-recall condition, and were probed for recall of the fictitious animal image/name pairs immediately after having been presented the pairs. The probe period in the immediate-recall condition was identical to that in the distractor-task condition. During fMRI testing, subjects completed the distractor-task condition twice, the immediate-recall condition once, and an “independent” version of the vocabulary task twice. The independent vocabulary tasks were identical in form to the distractor vocabulary task within the distractor-task condition, the only distinction being that the independent tasks were not preceded by an animal presentation period and were not followed by an animal probe period. The distractor-task condition and independent vocabulary task each occurred twice to provide additional data on which to train and test a pattern classifier. The immediate-recall condition occurred only once in order to reduce scan time and subject fatigue, as data from this task was not used for pattern analysis. Additionally, a 60-second block of multiple-choice trivia questions (e.g., “Which planet has the longest day?”) were included as brief rest periods to foster engagement and positive affect, and to minimize potential fatigues effects on the experiment tasks. Before each task and at the end of the run there was a 15-second fixation period during which subjects attended to a fixation cross. Task order was counterbalanced across subjects.

Each subject completed the tasks in one of the following two orders: distractor-task condition, independent vocabulary task, immediate-recall condition, trivia, independent vocabulary task, distractor-task condition; or independent vocabulary task, distractor-task condition, immediate-recall condition, trivia, distractor-task condition, independent vocabulary task. Order was chosen by counterbalance for the first 10 subjects and using random assignment to conditions for the remainder of subjects: Randomization was used after the first 10 subjects so that the order of tasks in the present experiment and the additional learning experiment conducted during the scan session would be independent.

In addition to the paired-associates task, subjects also completed an additional, exploratory associative learning task (a variant of “fast mapping,” adapted from [21]) during a separate, counterbalanced functional magnetic resonance imaging (fMRI) run. Specifically, subjects viewed fictitious animals alongside real, known animals during encoding and answered yes/no questions about the fictitious animals. Given that this task was used for testing separate study hypotheses from those here, the results are not presented in this report.

### MRI Acquisition

Scans were acquired using a Siemens 3T Verio scanner equipped with a 32-channel head coil (Siemens AG, Erlangen, Germany). After functional localizer scans, an MP-RAGE sequence (176 sagittal slices, FOV phase = 256 mm, 256 x 256 in-plane matrix, TR = 3200 ms, TE = 409 ms, TI = 900 ms, 1-mm isotropic voxels, flip angle = 9°) was used for acquiring whole-brain anatomical scans. Functional images were acquired with a gradient-echo T2*-weighted echo-planar sequence (42 axial slices, FOV phase = 192 mm, 64 x 64 in-plane matrix, TR = 2500 ms, TE = 30 ms, 3-mm isotropic voxels, flip angle = 83°).

Two 528-volume (1,320-second) functional runs were collected in a single scanning session. One run included the two distractor-task conditions, one immediate-recall condition, two independent vocabulary tasks, and one block of trivia questions. The other run included a separate, exploratory learning task.

### Behavioral Data Analysis

To determine whether offline processing led to better recall on the paired-associates task, we compared the number of times subjects correctly identified animal image/name pairs during the probe period in the distractor-task and immediate-recall conditions with a two-tailed, paired-samples t-test. This tested whether there was any improvement in the distractor task condition compared to the immediate recall condition.

Additionally, to determine whether offline processing interferes with the online performance of the distractor task, the number of correct responses on the independent vocabulary tasks was compared to the number of correct responses on the distractor vocabulary tasks within the distractor-task condition using a two-tailed, paired-samples t-test.

### MRI Preprocessing

Preprocessing and analysis of the fMRI data were carried out using SPM8 (Welcome Department of Cognitive Neurology, London, UK) implemented in MATLAB (version R2011b; MathWorks, Inc., Natick, MA, USA) as well as custom MATLAB scripts. Functional images were realigned to the first image of the functional run, and the structural image was coregistered to the mean of the functional images and segmented into grey matter, white matter, and cerebrospinal fluid maps using the ICBM space template. The functional images were then spatially normalized to MNI space using the normalization parameters from the structural segmentation.

### Univariate Functional Localizer

After the preprocessing steps listed above, functional images were spatially smoothed with an 8-mm^3^ full-width at half-maximum (FWHM) Gaussian kernel. First-level statistical analyses were performed using a general linear model (GLM). A design matrix was fitted for each subject including all task conditions across all runs, with six motion parameters included as regressors, leaving fixation as an implicit baseline. Task conditions were modeled as epochs (see MRI Procedure for task lengths). In order to identify regions of interest (ROIs) involved in image/name pair encoding, we conducted a contrast of encoding in all paired-associates tasks > baseline. Single-subject contrast maps were then submitted to a second-level random-effects group analysis (thresholded at *P* < 0.05, familywise error- (FWE-) corrected, voxelwise; *k* voxels per cluster > 20).

### Multivariate Analyses

MVPA was conducted in four ROIs identified with the functional localizer using 15-mm (5-voxel) radius spheres around the peak voxel from each cluster, as well as anatomically defined left and right hippocampal ROIs as an exploratory *post-hoc* analysis. Our first analysis tested the hypothesis that offline processing is accompanied by local neural information in ROIs that is absent when offline processing is not occurring. For this purpose we trained a linear support vector machine (SVM) to discriminate between neural activity during the distractor-condition vocabulary tasks (when offline processing should be occurring) and neural activity during the independent vocabulary tasks (when offline processing should not be occurring).

For this analysis, functional images were not spatially smoothed and neural activity was not deconvolved with the hemodynamic time course. Separately for each ROI, we collected activation patterns during each of the vocabulary tasks. To account for hemodynamic lag, the first 4 volumes (10 seconds) of each 48-volume vocabulary task were excluded from analyses, resulting in task blocks of 44 volumes each. The resulting patterns were then centered around their means both within and across voxels prior to classification separately for each ROI. For the purpose of cross-validation, training was performed on data from one block of each type (i.e., one block of the distractor-condition vocabulary task and one block of the independent vocabulary task) and testing was performed on the remaining blocks (for a total of 44 × 2 = 88 training patterns and 88 testing patterns). Discrimination performance was then encoded as sensitivity (*d’*). Next, to identify ROIs in which discrimination performance was significantly above chance we submitted *d’* values from all subjects for each ROI to a one-tailed Wilcoxon signed-rank test (*d’* > 0), a non-parametric test suited for testing data that deviates from normality.

Similarly, in a second MVPA, which examined whether activity during encoding was more frequently classified as the distractor-condition or independent vocabulary task, we acquired classification performance for each subject in each ROI (i.e., the percentage of observations classified as distractor-condition) and then conducted one-tailed Wilcoxon signed-rank tests for each ROI to determine if classification performance was above chance level (50%).

## Results

### Behavioral Results

**Fig 2:**
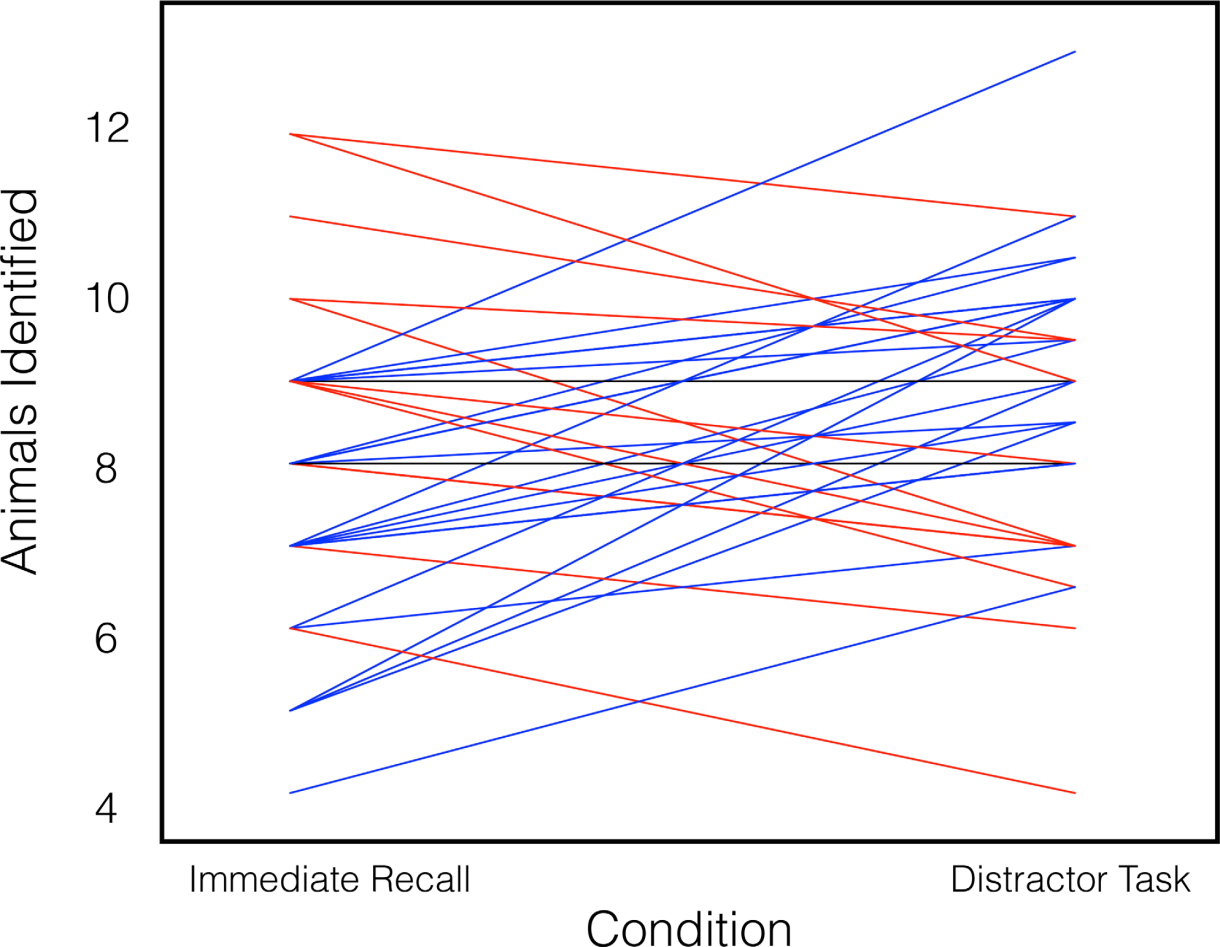
Behavioral performance in the two animal identification conditions. Each line represents performance of a single subject in each of the two conditions, which were performed in counterbalanced order. Subjects whose performance was superior in the immediate recall condition are plotted in red, subjects whose performance was superior in the distractor task condition are plotted in blue, and subjects whose performance was the same in the two conditions are plotted in black.

To determine whether offline processing improves behavioral learning performance, we compared performance after the distractor period (when offline processing would occur) with performance in the immediate recall condition, which allowed no opportunity for offline processing. As hypothesized, performance in the distractor task condition (mean number of animals identified = 8.73, standard deviation = 1.75; chance performance is 33% or 5 ⅓ image/name pairs) was superior to performance in the immediate recall condition (mean number of animals identified = 7.94, std. dev. = 1.88; paired-samples *t*-test; *t*(34) = 2.142, *P* = 0.040), demonstrating improved behavioral learning performance after the offline processing period (Fig 2).

To examine whether offline processing interferes with the simultaneous online performance of the distractor task, we compared performance between the distractor-condition and independent vocabulary tasks. There was no difference in accuracy (paired-samples t-test; *t*(34) = 0.729, *P* = 0.471) or reaction time (paired-samples *t*-test; *t*(34) = 1.091, *P* = 0.283) between the distractor-condition (mean accuracy = 7.41 / 15, std. dev. = 2.54; mean reaction time = 4.25 seconds, std. dev. = 0.68) and independent (mean accuracy = 7.16 / 15, std. dev. = 2.68; mean reaction time = 4.16 seconds, std. dev. = 0.57) vocabulary tasks, suggesting that offline processing does not significantly interfere with online performance of the distractor task.

### Regions Implicated in Offline Memory Processing

**Fig 3:**
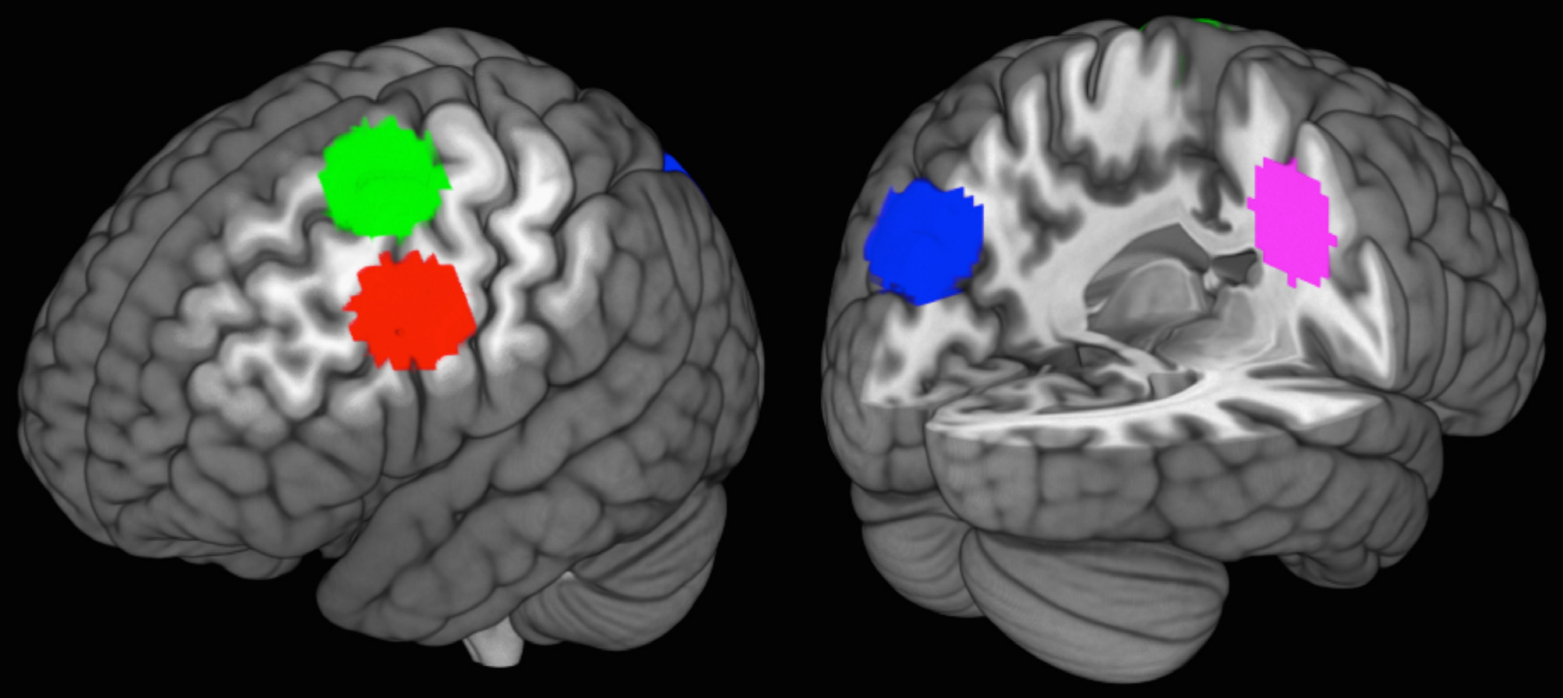
Cutaway volumes showing locations of ROIs identified with the encoding > baseline functional localizer. ROIs are 15-mm (5-voxel) spheres in left DLPFC (Brodmann area 9; –51, 8, 37; red), PrC (–18, –82, 40; blue), left middle frontal gyrus (–27, 2, 61; green), and right precentral gyrus (30, –7, 40; pink). DLPFC = dorsolateral prefrontal cortex; PrC = precuneus; ROI = region of interest.

To identify ROIs for use in multivariate analyses, we contrasted paired-associated encoding against baseline. This analysis identified clusters in PrC, left middle frontal gyrus, left DLPFC, and right precentral gyrus (Fig 3; Table 1), the peaks of which were used to define equal-volume ROIs for the purpose of our multivariate analyses. Specifically, spherical masks with radii of 15 mm were centered on these peaks for each ROI.

**Table 1:** Peak voxels, cluster sizes (k), and t-values for encoding-related neural activity (encoding > baseline). Peak voxels were used to define ROIs for the multivariate analysis. DLPFC = dorsolateral prefrontal cortex; PrC = precuneus; ROI = region of interest.

Region	Cluster peak (*x*, *y*, *z*)			Cluster size (*k*)	*t*-value
**PrC**	-18	-82	40	116	7.59
**Left middle frontal gyrus**	-27	2	61	118	6.98
**Left DLPFC**	-51	8	37	37	6.45
**Right precentral gyrus**	30	-7	40	107	6.43

**Fig 4:**
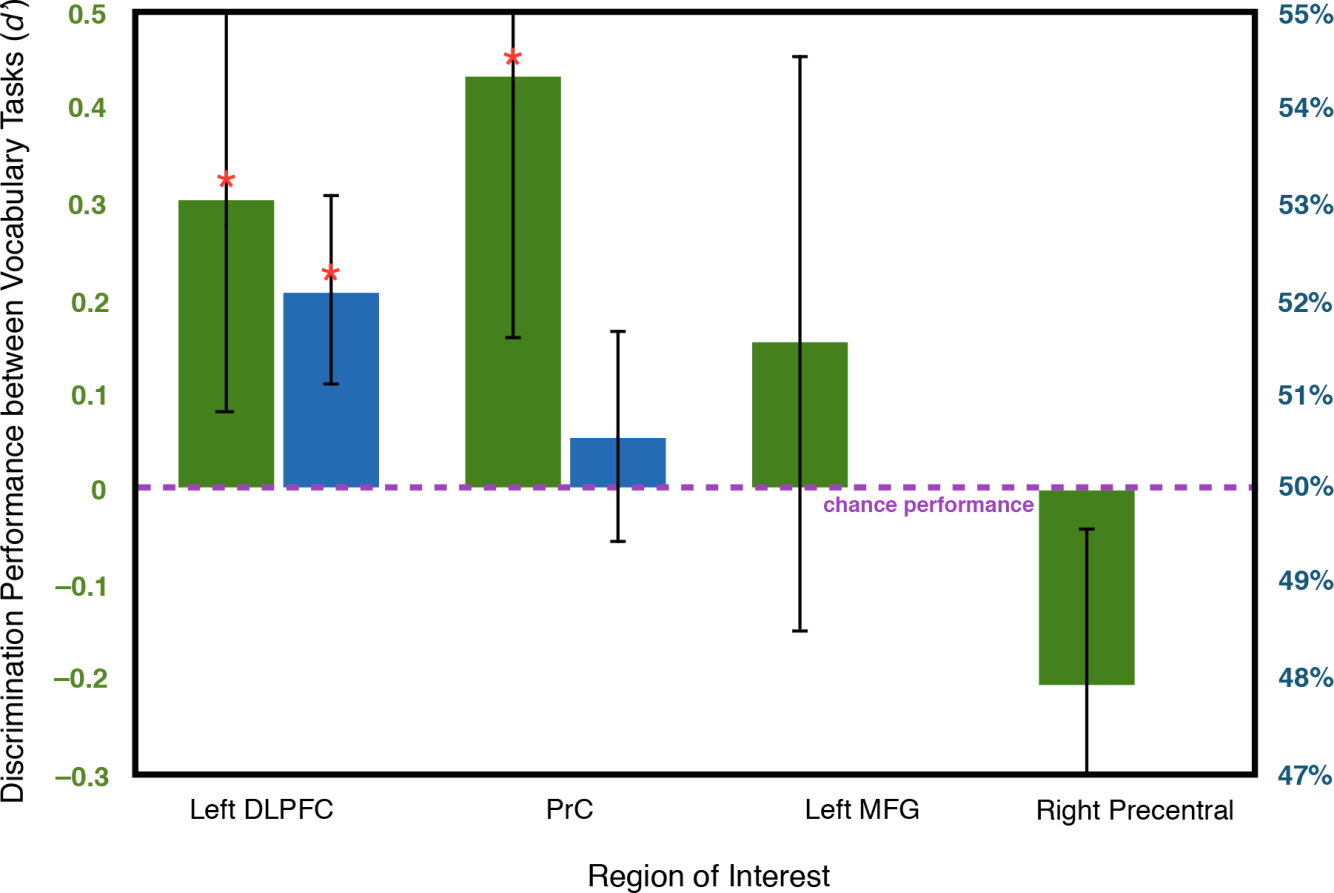
Classification performance in left DLPFC, PrC, left middle frontal gyrus (MFG), and right precentral gyrus in the multivariate analysis discriminating the distractor-condition and independent vocabulary tasks (left axis, green bars) and in the multivariate analysis classifying encoding activity as one of the two vocabulary tasks (right axis, blue bars). In the former analysis, classification performance was significantly better than chance in left DLPFC and PrC. In the latter analysis, encoding activity was classified as the distractor-condition vocabulary task significantly above chance frequency in left DLPFC. Note that only ROIs with above-chance classification performance in the first multivariate analysis were used in the second analysis. Error bars reflect standard error of the mean. DLPFC = dorsolateral prefrontal cortex; PrC = precuneus; ROI = region of interest.

The first multivariate analysis tested the hypothesis that local information related to offline processing of encoded image/name pairs would be detectable in the fMRI signal during the vocabulary distractor task in regions involved in encoding the pairs, specifically in DLPFC [9]. We trained a pattern classifier to discriminate between neural activity during the distractor-condition vocabulary tasks (when offline processing should be occurring) and neural activity during the independent vocabulary tasks (when offline processing should not be occurring). Indeed, both left DLPFC (Wilcoxon rank-sum *Z* = 1.99, *P* = 0.023) and PrC (*Z* = 2.50, *P* = 0.006) contained local information sufficient for a linear classifier to discriminate between the tasks, indicating that these ROIs are involved in offline processing (Fig 4). Local information contained in left middle frontal gyrus was marginally significant for discrimination (*Z* = 1.53, *P* = 0.063). The left and right hippocampal ROIs defined *post hoc* did not contain local information sufficient for discrimination.

After establishing that local information in ROIs indeed accompanies offline processing during the distractor task, we sought to answer whether this information reflects reactivated memory representations of encoded material in DLPFC. We trained the pattern classifier on the distractor-condition and independent vocabulary task activity and tested it on neural activity during image/name pair encoding in each ROI that was found to contain local information accompanying offline processing in the previous analysis. If the encoding-related neural activity in an ROI is more often classified as the distractor-condition vocabulary task, as opposed to the independent vocabulary task, this would suggest that local representations present during encoding are reactivated during offline processing. Consistent with this neural reactivation account, activity during encoding in left DLPFC was classified significantly more often as the distractor-condition vocabulary task than as the independent vocabulary task (52.0% overall classification as the distractor-condition task; Wilcoxon rank-sum *Z* = 1.89, *P* = 0.030).

### Temporal Proximity of Encoding and Vocabulary Tasks

An alternative explanation for our findings is that classification of encoding activity in left DLPFC as the distractor-condition vocabulary task merely reflected the closer average temporal proximity of the distractor-condition vocabulary task to the encoding period compared to the independent vocabulary task. To test for such temporal proximity or context effects, we looked for a correlation between proportion of classification as the distractor-condition vocabulary task and the volume number of the encoding period (i.e., time elapsed in the encoding period). If temporal proximity effects underlie the classification results, we would expect to see an increase in classification proportion of the encoding volumes as the distractor-condition vocabulary task throughout the encoding period, as the time between encoding and the distractor-condition vocabulary task decreases. We found no evidence of such a positive correlation between classification proportion and volume number of the encoding period (*r*(33) = –0.31, *P* = 0.964), indicating that temporal proximity is unlikely to explain the observed neural effects.

## Discussion

We demonstrate that a brief, active period of post-encoding offline processing boosts associative learning. Specifically, subjects correctly identified more fictitious animal image-name pairs after performing a distractor task for two minutes than they did when no opportunity for offline processing was provided, signifying the first demonstration of absolute improvements in associative memory performance following offline processing. Consistent with predictions regarding the neural underpinnings of this improvement, multivariate analyses revealed that during the distractor period left DLPFC and PrC contained information associated with the offline processing of learned material. Further, it was observed that activity patterns present in left DLPFC during encoding are reinstated during offline processing, supporting the hypothesis that encoded memory representations were reactivated during offline processing.

DLPFC has previously been shown to play a role in paired-associate encoding and retrieval processes [18,19,22–24], and has been theorized, along with ventrolateral prefrontal cortex (VLPFC), to execute control processes during declarative encoding [18], with DLPFC supporting relational processing [23] and VLPFC supporting selection processes [22]. Our findings extend this view of DLPFC function and suggest that processing of relational representations of stimulus pairs in associative learning may continue beyond encoding, albeit offline and without conscious control, in service of promoting memory for the associated stimuli.

PrC has been implicated broadly in episodic memory encoding [25–28] and retrieval [29], sometimes in concert with left lateral PFC [30]. Although little is known about the specific contributions of PrC to these processes, its recruitment during encoding appears to be greater when encoded material is associative in nature [31,32] as is the case in the present study. This associative recruitment makes sense in light of recent work identifying PrC as a connectivity hub, receiving input from and projecting to a number of other cortical locations [33–35]. In light of such findings, we speculate that PrC may support continued associative processing during the distractor period, potentially in tandem with DLPFC. However, additional research on the role of PrC in offline processing should be complemented by a more complete understanding of the PrC’s role in other processing domains [31].

Anatomically defined left and right hippocampal regions did not contain information sufficient for discrimination between distractor-period and independent vocabulary tasks. This may be partly attributable to weaker MRI signal in subcortical compared to cortical regions. It is also possible that processing in the hippocampus during encoding and subsequent offline processing may be sufficiently different that local patterns do not persist between these period that allow discrimination with the present methods. Future work should include paradigms and analytic approaches optimized for probing the role of the hippocampus and other medial temporal lobe structures in offline processing [13].

The present work in the associative learning domain contributes to a growing body of literature on offline processing and post-encoding memory reactivation [1–3,7–10,13,14,36] insofar as it demonstrates overall associative memory improvement following awake, offline processing and identifies a prefrontal region that appears to support reactivated memory representations. The present finding of awake, offline declarative memory enhancement stands in contrast to previous studies that failed to show such effects [1] but in line with others [5,9]. It is unclear what accounts for these discrepant findings in various studies, but we speculate that an interaction between the type of information to be remembered and the content of the distractor task may play a major role [1,4]. Given that research on offline processing remains a relatively young discipline, seemingly incongruous results should not be wholly surprising, and it will be the task of future work to resolve these incongruities. From the present results we conclude that the use of a challenging distractor task illustrates that offline processing can occur simultaneously with not just a simple, active task [13], but also with a task requiring considerable attention and engagement (i.e., a challenging vocabulary task). This observation places constraints on potential neural systems and cognitive operations involved in offline processing, as their activity must not preclude the simultaneous performance of a complex, attention-demanding task. Such inferences illustrate the value of considering multimodal evidence to shape the hypothesis space and determine boundary conditions of offline processing.

Although a number of studies have demonstrated that periods of offline processing during both sleep and wakefulness can stabilize and enhance memories [1–3], demonstrations of awake enhancement of declarative memory have been rare in the literature, and the present study signifies the first demonstration of awake enhancement of associative material, and thus replications of this finding will be needed. Declarative memory improvement following sleep [37] and wakeful rest [5] have previously been demonstrated, but the present study’s demonstration of memory improvement following a distractor task is noteworthy in that it suggests that sleep or wakeful rest are not necessary for the occurrence of memory enhancement as a result of offline processing, but rather that offline processing may occur simultaneously during active, goal-directed cognition.

The behavioral improvement reported in the present study supports the hypothesis that offline processing is involved in a broad array of functions relevant to declarative memory beyond mere memory stabilization [1,9]. At the same time, we note that to further characterize awake declarative memory enhancement it will be necessary to explore its prevalence in declarative learning paradigms different from the one reported here. In particular, the two-minute offline processing period in the present experiment was more than an order of magnitude shorter in duration than those typically employed in memory research (hours to days [1–3,7,8]), suggesting that offline processing may influence behavioral measures of memory and learning much more rapidly than previously believed.

The present findings may also shed light on well-known phenomena such as the contextual interference effect [38]. This effect is characterized by improvement in motor skill acquisition when the acquisition period is broken up by performance of unrelated tasks. It is possible that offline processing occurs during the unrelated tasks in such paradigms, similar to the offline processing during the distractor task in the present study. If similar cognitive mechanisms indeed underlie these phenomena, the present study suggests potential neural mechanisms of contextual interference, as well.

A potential alternative explanation for the finding that neural activity in DLPFC during encoding was more similar to activity during the distractor-condition than during the independent vocabulary task is that the distractor-condition vocabulary task always immediately followed the encoding period, whereas the independent vocabulary task either immediately preceded encoding or followed the distractor-condition vocabulary task. The independent vocabulary task thus was sometimes more temporally distant from encoding than the distractor-condition vocabulary task. This would suggest that effects not related to offline processing, such as context effects or scanner drift, account for the greater apparent similarity of encoding-period activity to distractor-condition vocabulary task activity. Our analysis of the timecourse of encoding pattern similarity does not support this interpretation, as the longitudinal increase in classification of encoding activity as distractor-condition activity that this interpretation would predict was not observed.

The analytic approach employed in the present study may appear backward compared to what one might expect when testing whether representations present during encoding reactivate during a subsequent distractor period. While we note that an approach involving training a classifier on neural activity present during encoding and then testing it on activity during a distractor task may be more straightforward and intuitive (e.g., [13]), the present approach answers the same question, namely whether activity patterns are shared between encoding and the distractor-condition vocabulary task. Given our desire to include both distractor-condition and independent vocabulary tasks in the design in order to compare performance between the two, the present analytic approach was well-suited. As pattern classification approaches are increasingly employed to investigate offline processing and memory reactivation, the existence of a broad array of potential designs and approaches will be desirable [20], and it is our hope that the one employed here will be useful in future studies of offline processing.

Critical to the claim that the present experimental manipulation evoked offline processing during the vocabulary distractor task is the assumption that subjects were not systematically engaging in online (conscious) rehearsal of encoded material during the distractor task. Though it is not possible to rule out this interpretation with complete certainty, we did not find evidence to support it: There were no differences in either accuracy or reaction time when comparing the distractor-condition and independent vocabulary tasks [9], as would be expected if subjects were consciously rehearsing animal image/name pairs during the distractor-condition vocabulary task. It is noteworthy that previous studies have often relied on unconstrained wakefulness [4,39–41] or passive rest periods [5] in investigating offline processing, allowing little control over subjects’ potential online rehearsal of learned material. In contrast, our use of a difficult distractor task makes conscious processing difficult and provides a way to indirectly measure, via performance effects, the occurrence of conscious processing of encoded stimuli. That said, future work will need to more precisely characterize subjects’ conscious thoughts during the distractor period. This is a challenging problem because more direct methods of monitoring, such as experience sampling, are likely to interfere with the distractor period and with offline processing. Additionally, it remains possible that subjects do experience conscious thoughts about or actively rehearse learned material, but that this is not the driver of the presently observed memory improvement.

Similarly, it is possible that fatigue contributed to the effects observed in the present experiment, with the distractor task providing an opportunity for recovery from fatigue and leading to improved memory for stimuli. We note that past work [9,42] has utilized conditions other than a distractor task following encoding and shown that a distractor task preferentially leads to improved performance on the following probe. Other work [43] has shown that a subject’s knowledge that a test (in that study, a decision making problem) will follow a distractor period is critical for performance improvements, suggesting that fatigue is not the driving factor for behavioral improvements, as fatigue is likely similarly diminished by a distractor task regardless of knowledge of an upcoming test. Nonetheless, we cannot definitively exclude fatigue as an explanation for the present results, and future work should include explicit controls for effects of fatigue.

Finally, we note that the present work raises a number of questions and potential directions for future studies of offline processing and its contributions to learning and memory more broadly. First, the biological mechanisms underlying the memory improvement observed in the present study are not known. Although the present study identifies a neural region that may contain reactivated memory representations during offline processing, the specific computations and neural operations occurring within this and other regions in support of offline processing remain obscure. To this end, stronger links should should be established between the study of such processing in humans and in other mammals where contrasting methodologies are used. It may be the case that on a cellular level the reactivated representations we measure in the human brain via fMRI are related to neuronal activity observed during neural replay in rodents [36], or that the cellular and synaptic mechanisms involved in human awake, offline processing are related to those identified in recent studies of memory consolidation and enhancement in animals [44]. Much additional work will be required for such mechanistic characterization of the present phenomenon. Second, it is unclear whether and how the specific content of the distractor task influences offline processing. It is possible that the memory enhancement observed here would have occurred during any of a variety of active or passive task conditions, and that the nature of the distractor task itself has little impact. Alternatively, there may be a relationship between specific distractor tasks and the material being processed offline, with some distractor tasks facilitating or enabling offline processing and others disrupting it [1,45]. The existence of such a relationship would have implications for models of interacting memory systems in offline processing effects [1,46]. Third, as mentioned above, further research on the temporal dynamics of memory reactivation during offline processing is needed: It is not known whether the two-minute distractor period used in our study falls near the minimum duration at which behaviorally significant offline processing may still occur. It remains possible that even shorter durations would still lead to measurable behavioral effects. Fourth, to aid in our understanding of memory-related offline processing, stronger links should should be established between the study of such processing in humans and in other mammals where contrasting methodologies are used. Finally, we believe that the present work is relevant to educational approaches and education research, and we hope that future studies will explore potential applications of offline learning in real-world learning contexts, such as in the classroom.
